# Construction of a semi-automatic ICD-10 coding system

**DOI:** 10.1186/s12911-020-1085-4

**Published:** 2020-04-15

**Authors:** Lingling Zhou, Cheng Cheng, Dong Ou, Hao Huang

**Affiliations:** Department of Information, Daping Hospital of Army Medical University, 10 Changjiang Access Road, Chongqing, 400042 China

**Keywords:** ICD-10 coding, Automatic coding-description models of the regular expressions - diagnosis codes - diagnosis descriptions

## Abstract

**Background:**

The International Classification of Diseases, 10th Revision (ICD-10) has been widely used to describe the diagnosis information of patients. Automatic ICD-10 coding is important because manually assigning codes is expensive, time consuming and error prone. Although numerous approaches have been developed to explore automatic coding, few of them have been applied in practice. Our aim is to construct a practical, automatic ICD-10 coding machine to improve coding efficiency and quality in daily work.

**Methods:**

In this study, we propose the use of regular expressions (regexps) to establish a correspondence between diagnosis codes and diagnosis descriptions in outpatient settings and at admission and discharge. The description models of the regexps were embedded in our upgraded coding system, which queries a diagnosis description and assigns a unique diagnosis code. Like most studies, the precision (P), recall (R), F-measure (F) and overall accuracy (A) were used to evaluate the system performance. Our study had two stages. The datasets were obtained from the diagnosis information on the homepage of the discharge medical record. The testing sets were from October 1, 2017 to April 30, 2018 and from July 1, 2018 to January 31, 2019.

**Results:**

The values of P were 89.27 and 88.38% in the first testing phase and the second testing phase, respectively, which demonstrate high precision. The automatic ICD-10 coding system completed more than 160,000 codes in 16 months, which reduced the workload of the coders. In addition, a comparison between the amount of time needed for manual coding and automatic coding indicated the effectiveness of the system-the time needed for automatic coding takes nearly 100 times less than manual coding.

**Conclusions:**

Our automatic coding system is well suited for the coding task. Further studies are warranted to perfect the description models of the regexps and to develop synthetic approaches to improve system performance.

## Background

The International Statistical Classification of Diseases and Related Health Problems (ICD) [1] is the foundation for the identification of health trends and statistics globally and the international standard for reporting diseases and health conditions. The ICD has been published in a series of editions to reflect advances in health and medical science over time. The 10th version, ICD-10, was endorsed in May 1990 and is used by more than 100 countries around the world. In China, ICD-10 was adopted in 2001 by the Peking Union Medical College Hospital of the World Health Organization (WHO) Collaborating Centre for the Family of International Classifications. The Chinese Version of Classification and Codes of Diseases (CCD) (GB/T 14396–2001), is an expanded version of ICD-10 and is widely used in China. In 2011, the National Health and Family Planning Commission of the People’s Republic of China promulgated the notice on using CCD codes for encoding diagnoses in the medical records after January 1, 2012 [2]. However, the vast area and large population of China have caused increases in numerous local diseases and special diseases, resulting in the localized ICD-10 coding systems have been widely explored by health departments of various provinces and cities. As a military hospital, we adopt the uniform coding rules based on the ICD-10 issued by the military management department, and add some additional codes according to the actual situation of our hospital. Although a version of the ICD-11 code set was released on June 18, 2018 to allow Member States to prepare for its implementation by the WHO, our military unit is still committed to using the ICD-10 to lay the foundation for using ICD-11.

Diagnosis-related group (DRG) is defined as classifications of each patient according to the diagnosis and other characteristics of the case. Diagnosis-related group-based prospective payment system (DRG-PPS) is one type of advanced hospital payment reimbursement mechanism. Since the 1990s, payments based on DRG have gradually become the principal means of reimbursing acute inpatient care in most high-income countries [3, 4]. Approximately a decade later, more and more low- and middle-income countries have begun to establish DRG-based payment systems. In July 2011, the pilot project of DRG was launched in 6 hospitals in Beijing, China. On June 2, 2017, the National Health Commission of the People’s Republic of China held a pilot meeting on DRG-PPS reform in Shenzhen, Guangdong Province. The reform of payment systems in public hospitals has entered a crucial stage in our country. The most commonly used diagnosis classification standard in DRG is the ICD-10 code system, and the quality of coding based on ICD-10 codes is directly related to the DRG grouping, which directly affects the economic benefits of the hospital.

Manually assigning ICD codes is expensive, time consuming and error prone due to the vast coverage and extent of ICD terminology. Many scientists have explored automatic and semi-automatic ICD coding as a solution to the massive amounts of human labour required for manual coding. For example, several studies based on machine learning approaches, such as the support vector machine (SVM) method [5–8], were proposed to automatically assign ICD-10 codes. With the extensive application of deep learning methods in various fields, these methods have also been widely used in automated ICD coding [9–12]. These studies indicate that deep learning models can produce interpretable results and can code automatically in a reasonable way. Meanwhile, studies [9, 10] also indicate that the deep learning framework outperforms SVMs. Other techniques based on natural language processing (NLP) [13–17] can significantly increase the performance of automatic ICD coding by mapping the already assigned diagnoses of patients to ICD codes. Study [18] used word embedding combined with a convolutional neural network (CCN), which showed outstanding performance compared with the NLP plus supervised machine learning models. A prior study [19] automatically classify patients’ diseases into an ICD-10-CM category based on the well-known Web Ontology Language. Another study [20] combined three coding systems into a single superior system to improve the performance of automated ICD-9-CM codes in clinical reports. In [12, 21, 22], ICD-10 coding of death certificates has already been addressed by automation. In [23] semi-automatic assignment of the indexing system was performed by exploiting the idea of the bag-of-words mapping indexing tool. Most of the above methods are only theoretical, and they have not yet been applied in practice.

At present, there are mainly two ways of coding in our country. One way is that clinicians are required to learn the ICD-10 codes and enter the clinical diagnosis with the help of a navigation tool within the nomenclature according to the coding principle. Clinicians completing patient records can use this tool to obtain a preliminary coding of the record, and then the medical record coders perform the quality audit. The other way of coding is that the medical record coders code according to the clinician's diagnosis records by using the computer dictionary library and then communicate with the clinicians when disputes arise. The first method has a higher coding efficiency, and the other method has a higher coding quality. The first method increases the workload of clinicians and requires better information technology for hospitals, so ICD-10 coding in many hospitals is still undertaken by the coders in medical records departments according to the free text form in the clinician's diagnosis records. Our hospital also adopts the second method. There are several types of errors in this method. First, clinicians often utilize abbreviations or synonyms, which causes imprecision and ambiguity when the coders are matching ICD-10 codes to these diagnosis descriptions. Second, several diagnosis descriptions are closely related and should be combined in many cases into a single combination ICD-10 code. However, coders may code each disease separately. Third, ICD-10 codes are organized in a hierarchical structure where the top-level codes represent generic disease categories and the bottom-level codes represent more specific diseases [24], and the coder may match the diagnosis description to a generic code instead of a specific code. In addition, the coders could make obvious errors due to carelessness when the workload is heavy.

There is a limited set of diseases that hospitals can treat. Different clinicians have their own recording habits. Through statistical analysis, their regularity can be determined. The ICD coding set has clear coding rules that are suitable for clustering and matching by related technologies. To improve coding efficiency and quality and to reduce coding errors, we aim to build an automatic ICD-10 coding machine on the basis of the existing coding system, which translates the free-text diagnosis descriptions into ICD-10 codes.

## Methods

### Coding system

Our hospital, as a member of a large-scale comprehensive medical institution in Chongqing, China, is involved in medical care, preventive health care, education and scientific research. The annual average number of discharged patients is approximately 100,000. A large number of discharge medical records are waiting to be coded by coders. The coding system-we currently use is called the Medical Record Cataloging System–a subsystem of the hospital information system. It consists of general sociodemographic information, diagnosis information, surgical information, and cost information. All of the above information forms the homepage of the discharge medical records. The general sociodemographic information is filled in by the staff at the admissions office when the patient is admitted, while the cost information is completed by the staff of the cost office when the patient is discharged from the hospital. Diagnosis information includes the diagnosis descriptions of outpatients, admissions, discharges, and their corresponding diagnosis codes, treatment results, and treatment days. Operation information includes the operation descriptions, operation codes, surgeons and anaesthesiologists, and operating date. After clinicians record these descriptions, the coders complete the corresponding codes with the aid of the ICD-10 dictionary library embedded in the Medical Record Cataloging System. In this study, only diagnosis descriptions and corresponding diagnosis codes were used.

### Constructing the upgraded coding system

The description models of the regular expressions (regexps) were applied to automatically transform the diagnosis descriptions to the matching codes in the upgraded coding system. The following steps specifically describe how to build the system.

Step 1: Data pre-processing. The diagnosis descriptions of the modeling datasets are classified based on the diagnosis codes. When the same diagnosis description corresponds to multiple diagnosis codes or multiple diagnosis descriptions correspond to one diagnosis code, the one that most coders agree on is used. According to the ICD-10 coding guidelines, each diagnosis code matches a diagnosis description.

**Fig. 1:**
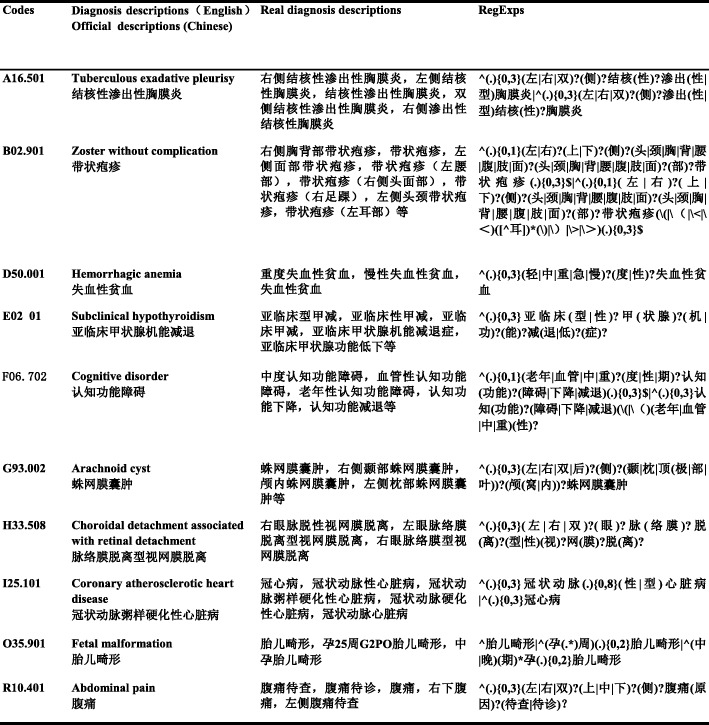
Examples of Diagnosis Descriptions Transformation into RegExps. The table shows the description models of the regexps were constructed by special symbols joining diagnosis descriptions. A diagnosis can be described by many different Chinese words, so we use Chinese to better present the application of regular symbols. This modeling method is also applicable to other languages

Step 2: Diagnosis terminology parsing. The nonstandard diagnosis descriptions representing the same disease recorded by clinicians were transformed to a diagnosis description through the description models of the regexps. A description model of the regexps matches an ICD-10 code. The regexps [25] replace the usual percentage (%) and other similarity lookup methods by using some pre-agreed combination of regular special symbols, such as ^, *,., x|y, or?, as shown in Table 1. Each special symbol represents a different meaning and is combined into the regexps. For example, complete abortion, foetal malformation, abdominal pain, haemorrhagic anaemia, ect, were translated into regexps, and we show these in Chinese due to the diversity of diagnosis descriptions recorded by clinicians in our country (Fig. 1). All the description models of the regexps were generated manually by our information engineers in cooperation with the coders. A rule base consists of these established description models of the regexps is used for subsequent program running.

**Table 1:** Examples of Regular Special Symbols and Meaning

Regular symbols	Meaning
**^**	Start position of string
*	The front character or expression 0 or more times.
**.**	Any single character other than null
**x|y**	X or Y, where x and y are one or more characters
**?**	Match the previous sub-expression zero or once

Step 3: Automatic coding. Diagnosis descriptions waiting for coding after the clinician fills in the homepage are automatically stored in Oracle Database 10 g Software [26]. The REGEXP_LIKE function supported by Oracle [27] is used to complete the one-to-many matching between the transformed diagnosis descriptions and an ICD-10 code. If the coding matching is completely consistent, the diagnosis code is automatically generated, and the coding log is updated. If the diagnosis description cannot be matched or corresponds to multiple regexps, the coding failure log is generated.

Step 4: Code auditing. The coders check the coincidence of the automatically completed diagnosis codes one by one through the Medical Record Cataloging System and process the failed codes, including repeated codes, mismatch codes and loss codes. These errors are fed back to the programmer to modify the regexps. Eventually, the regexps are gradually optimized to reduce system coding errors.

**Fig. 2:**
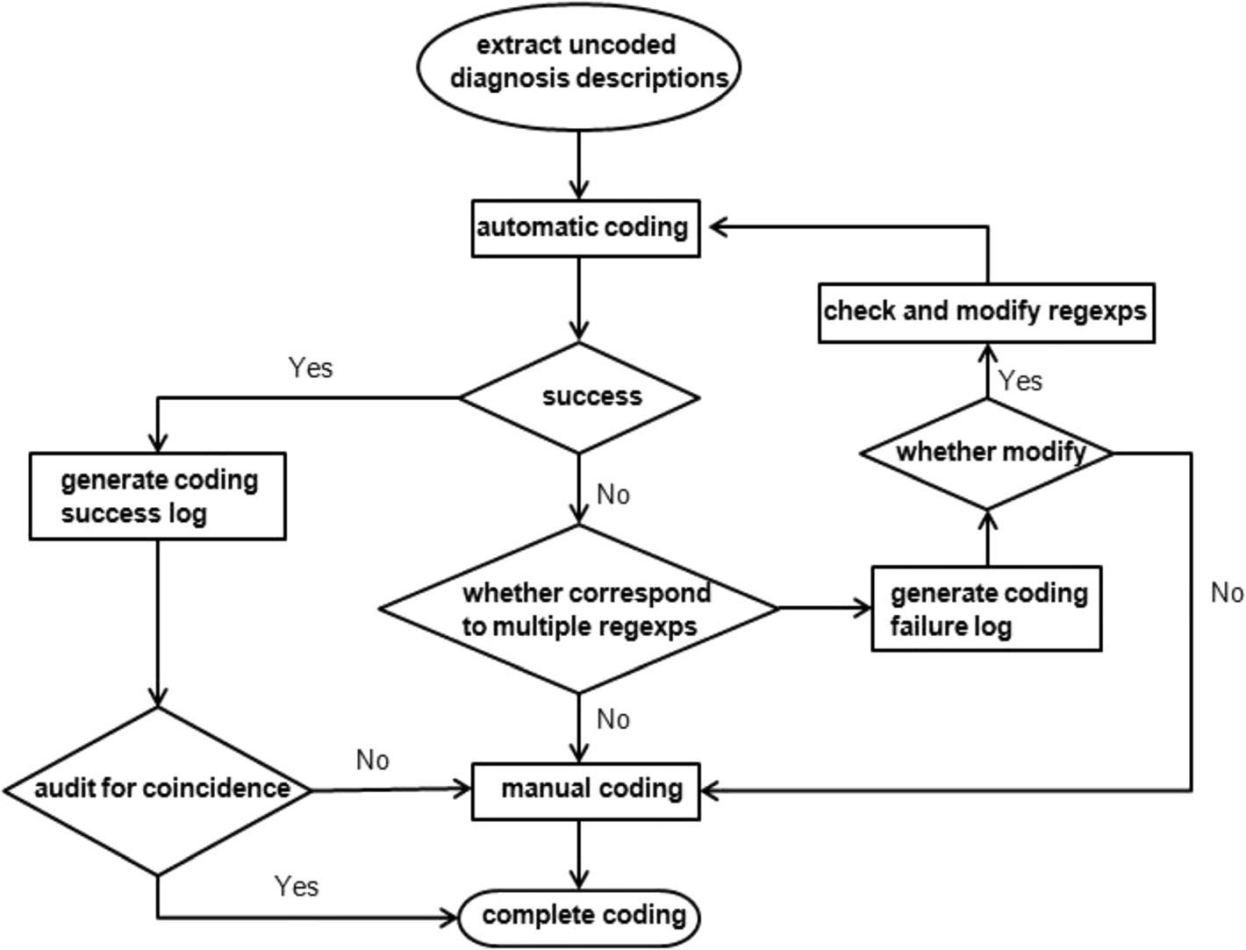
The flow chart of automatic coding with ICD-10. The process from waiting for coding to complete coding

The flow chart of automatic coding is shown in Fig. 2. Procedural codes and structured query language (SQL) statements are shown in Additional file 1.

### Datasets

Our datasets were obtained from the diagnosis information on the homepage of the discharge medical records. The study consisted of two stages, which were divided according to the coding date and time. In the first stage, the top 500 high-frequency diagnosis code categories from January 1, 2014 to June 30, 2017 were used to establish the description models of the regexps. Modeling and optimization lasted 3 months. The test performance between October 1, 2017 and April 30, 2018 proved that the first phase of the study was effective. Therefore, further research was carried out to increase the total number of matching code categories (up to 1000) in the second stage. The other unmatched (in the first stage) top 500 high-frequency codes between January 1, 2014 and April 30, 2018 were modeled. The second phase of the experiment lasted 2 months. Testing results from July 1, 2018 to January 31, 2019 were used to further suggest the effectiveness of this study.

### Evaluation metrics

System performance [20, 28] was evaluated using precision (P), recall (R), the F-measure (F) and overall accuracy (A). P is defined as the ratio of true positive (TP) to the total number of TP and false positive (FP). R is the ratio of TP to the total number of TP and false negative (FN). F represents the harmonic mean of the precision and recall, where α is set to 0.5, indicating that equal weight is given to the precision and recall. A is the fraction of coding results assigned correctly among all the codes (TN represents true negative). The definition and equations are shown in Table 2 and Eqs. 1–4.

1 $$ P=\frac{\mathrm{TP}}{\mathrm{TP}+\mathrm{FP}} $$

2 $$ R=\frac{\mathrm{TP}}{\mathrm{TP}+\mathrm{FN}} $$

3 $$ F=\frac{1}{\upalpha \frac{1}{\mathrm{p}}+\left(1-\upalpha \right)\frac{1}{\mathrm{R}}} $$

4 $$ \mathrm{A}=\frac{\mathrm{TP}+\mathrm{TN}}{\mathrm{TP}+\mathrm{FP}+\mathrm{TN}+\mathrm{FN}} $$

**Table 2:** The Definition of Evaluation Result

Display ICD-10	Condition positive	Condition negative
Automatically display	TP (Correct result)	FP (Unexpected result)
Doesn’t automatically display	FN (Missing result)	TN (Correct absence of result)

## Results

**Fig. 3:**
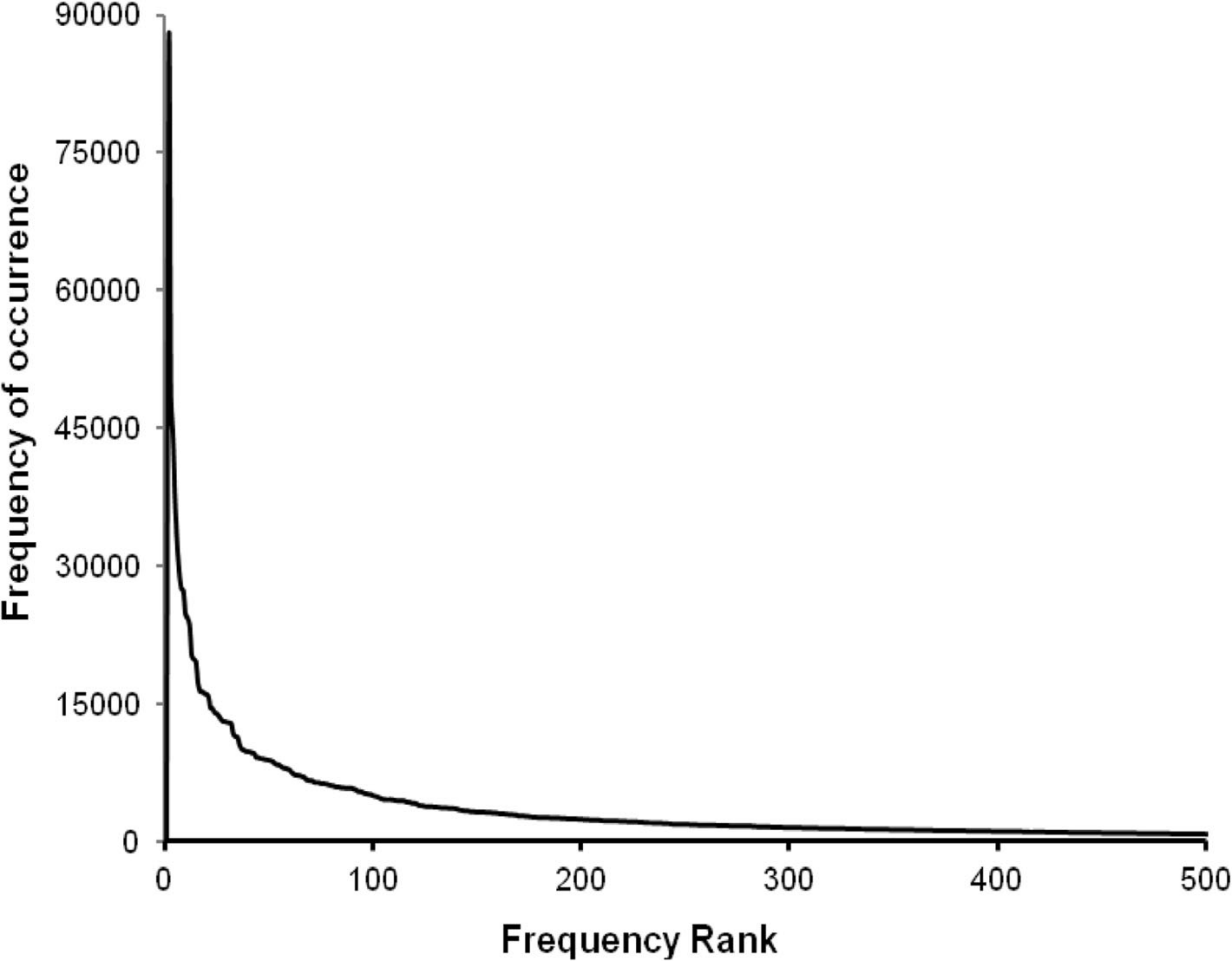
Distribution of the top 500 diagnosis codes. Horizontal and longitudinal ordinate represent the frequency rank and the frequency of occurrence, respectively, from January 1, 2014 to June 30, 2017

**Fig. 4:**
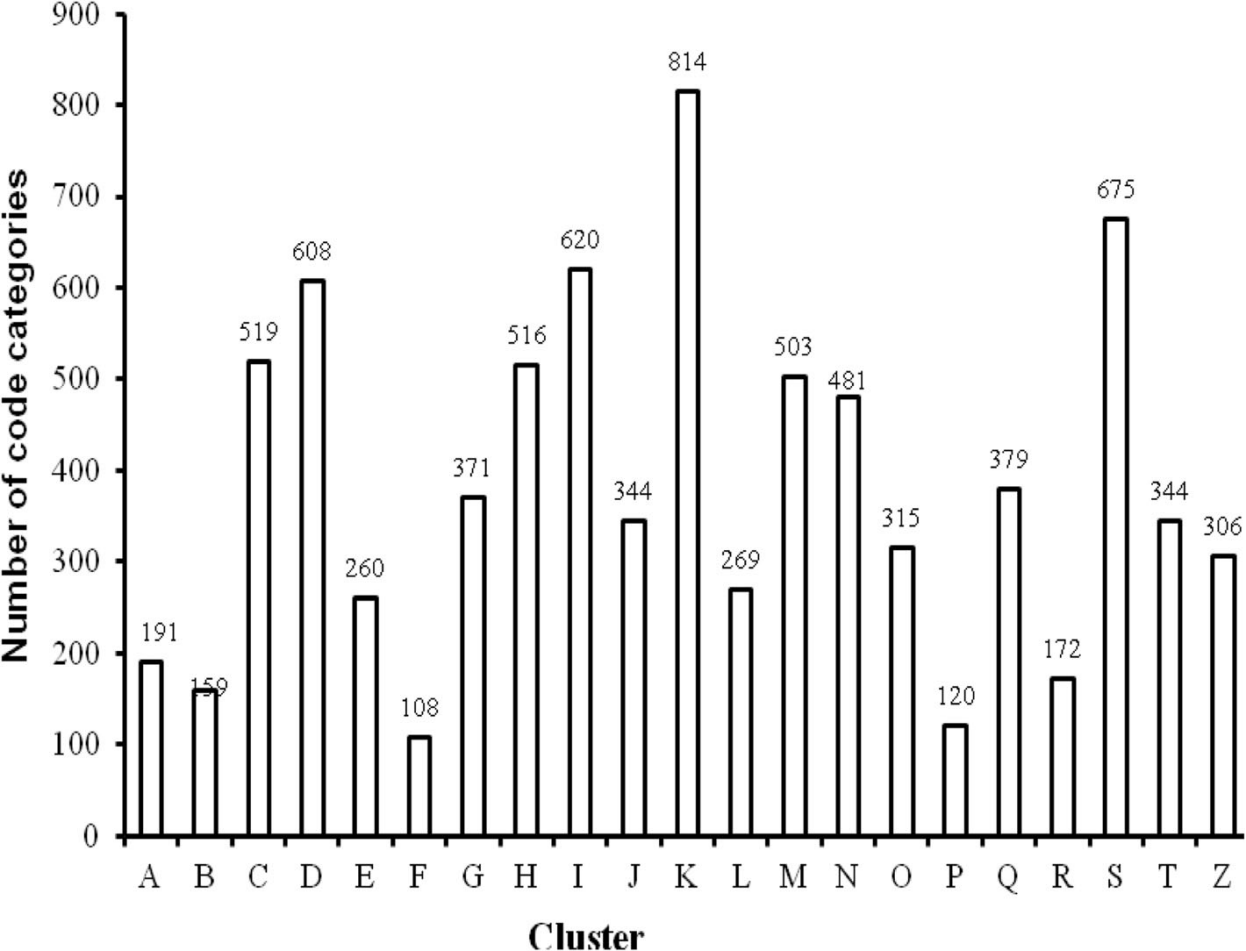
Number of code categories per cluster. Horizontal ordinate represents the each chapter of disease coding according to ICD-10. Longitudinal ordinate represents the number of code categories corresponding to each cluster from January 1, 2014 to June 30, 2017

**Fig. 5:**
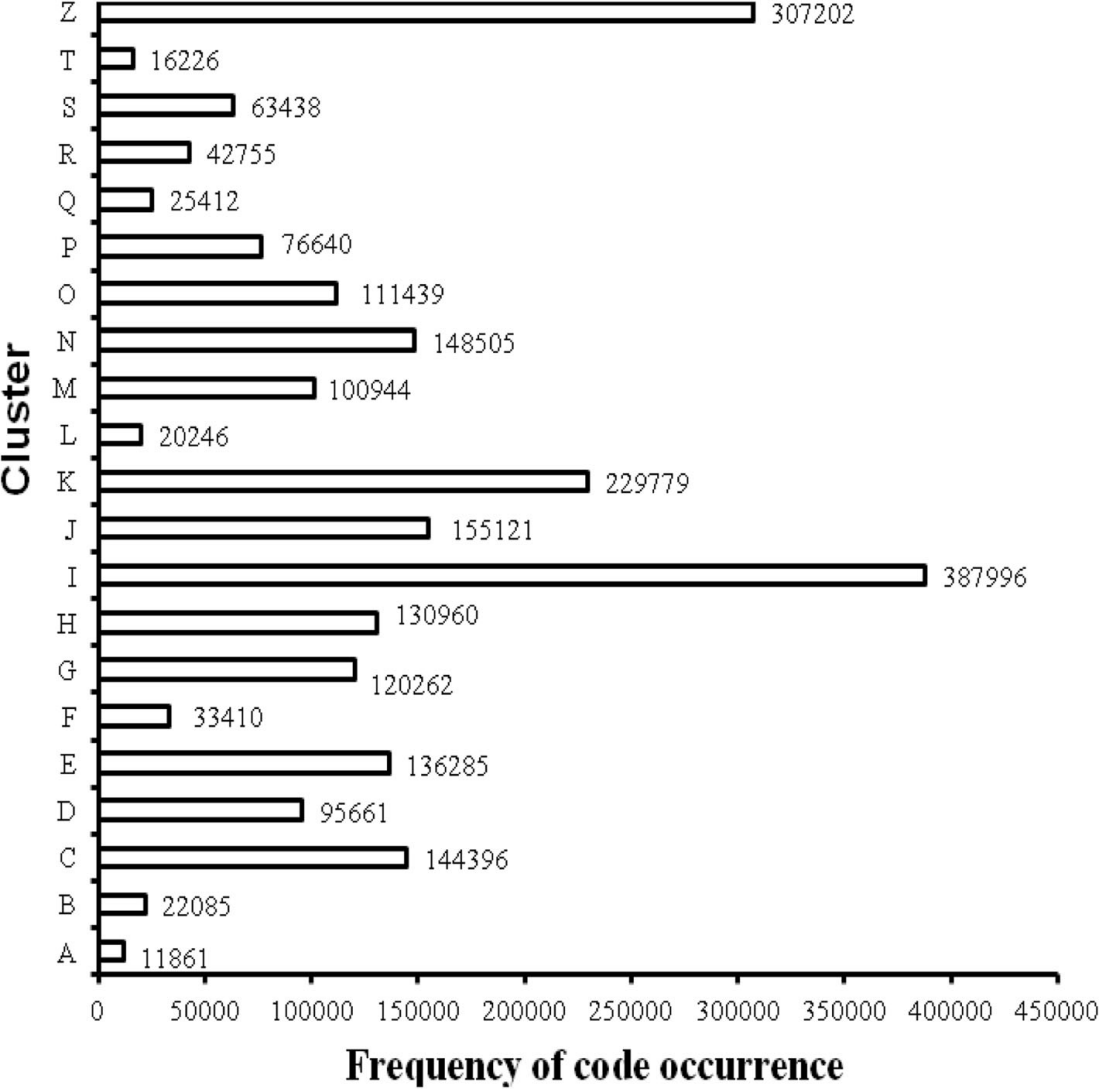
Number of diagnosis codes per cluster. Horizontal ordinate shows the frequency of code occurrence corresponding to each cluster from January 1, 2014 to June 30, 2017

The experimental results of the first stage are as follows. From January 1, 2014 to June 30, 2017, the total number of patients discharged was 383,440, with a total of 2,380,623 diagnosis codes and 8074 code categories (see Additional file 2). An average of 6 diagnosis descriptions per patient was coded by coders. Figure 3 shows the distribution of the top 500 diagnosis codes with high frequency, which indicates that the high-frequency codes are concentrated in the top 100. The top three codes were I25.101, I10 06 and E11.901, which appeared 87,008, 49,128 and 44,430 times and represent coronary atherosclerotic heart disease, essential hypertension grade III and type 2 diabetes mellitus, respectively. According to the ICD-10 classification, Fig. 4 shows the histogram of the number of code categories per cluster, which shows that class K contains the most code categories and class P contains the least. Figure 5 shows the histogram of the number of diagnosis codes per cluster, and class I contains the most codes (387,996). The evaluation results of the first test phase are presented in Table 3, which shows high precision.

**Table 3:** Evaluation Results of Two stage

Stage	Testing sets	TP	FP	FN	TN	P(%)	R(%)	F(%)	A(%)
First	10/1/2017–4/30/2018	50,084	6022	161,478	67,217	89.27	23.67	37.42	41.19
Second	7/1/2018–1/31/2019	85,838	11,291	222,938	82,994	88.38	27.90	42.41	41.89

**Fig. 6:**
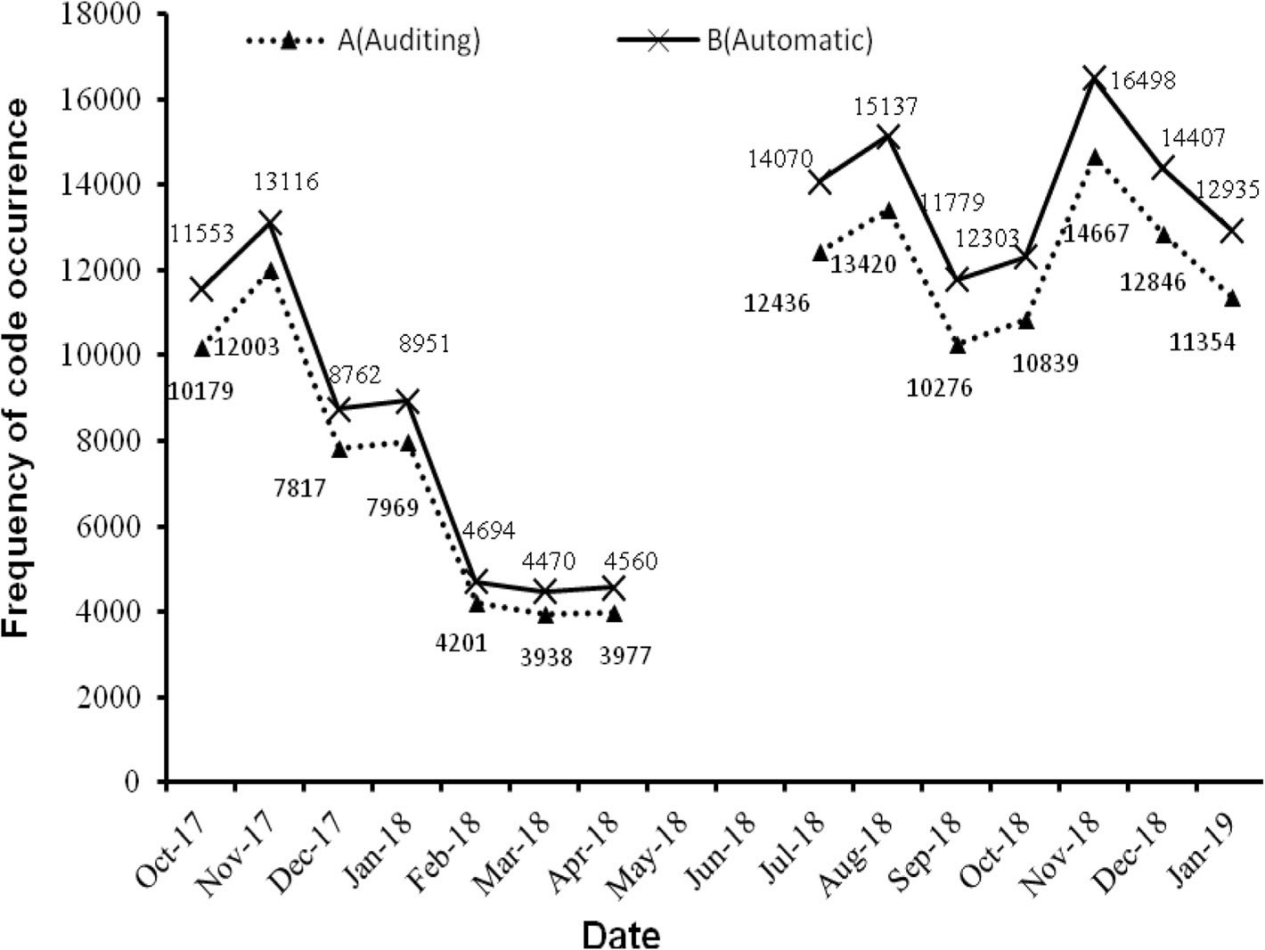
Comparison between the number of correct auditing codes and the automatic codes. Longitudinal ordinate shows the coding quantity per month from October 1, 2017 to April 30, 2018 and from July 1, 2018 to January 31, 2019. Curve A and B represent the correctly assigned diagnosis codes by the code auditors and the automatically completed codes by the coding system, respectively

**Fig. 7:**
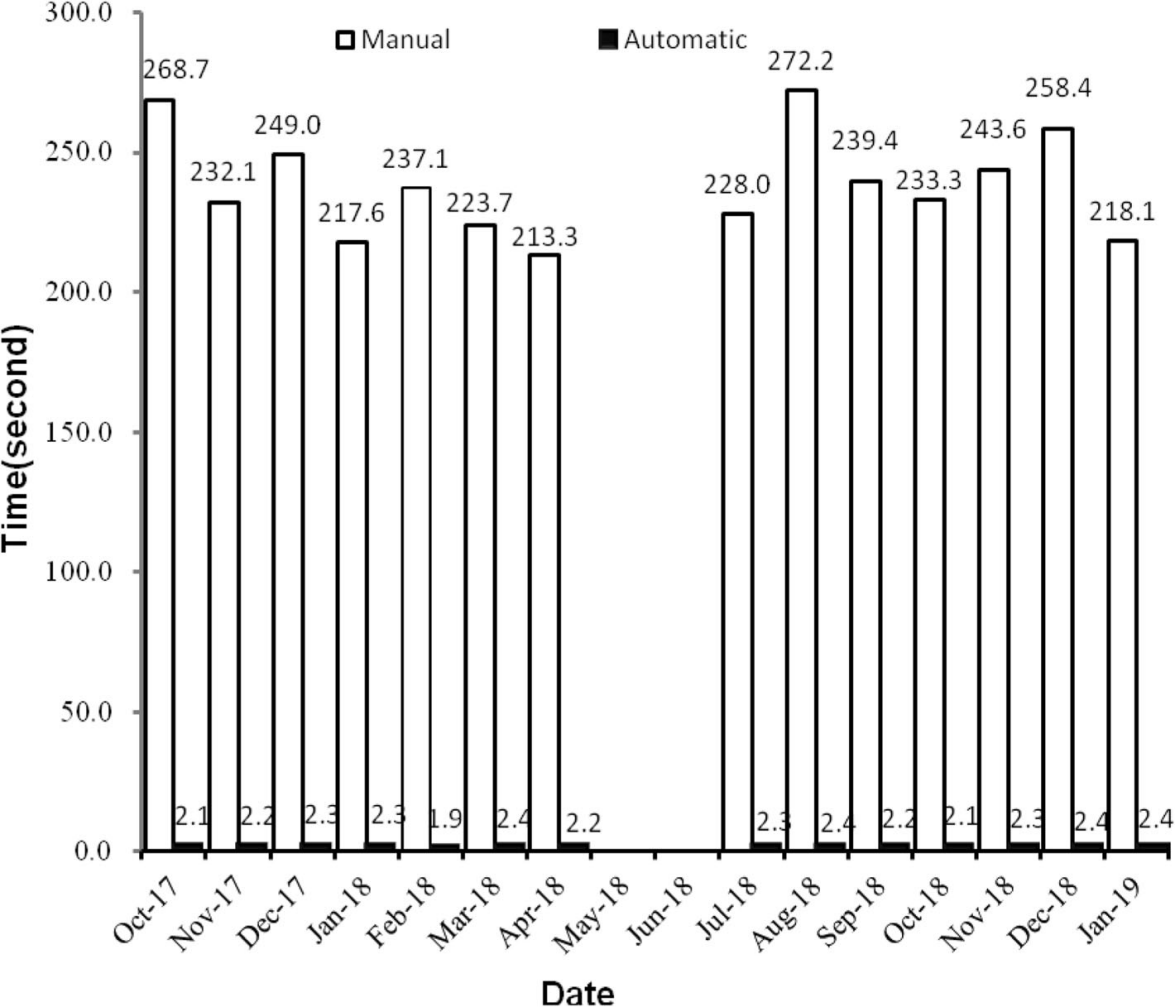
Time needed for manual coding and automatic coding. Longitudinal ordinate shows the average time-consuming (seconds) per ten codes every month from October 1, 2017 to April 30, 2018 and from July 1, 2018 to January 31, 2019

Figure 6 shows the monthly trends in coding quantity, and curves A and B represent the correctly assigned diagnosis codes by the code auditors and the automatically completed codes by the coding system, respectively, in every month of testing phase. The two curves are very similar, which indicates high precision. A comparison between the time needed for manual coding and automatic coding is shown in Fig. 7.

**Fig. 8:**
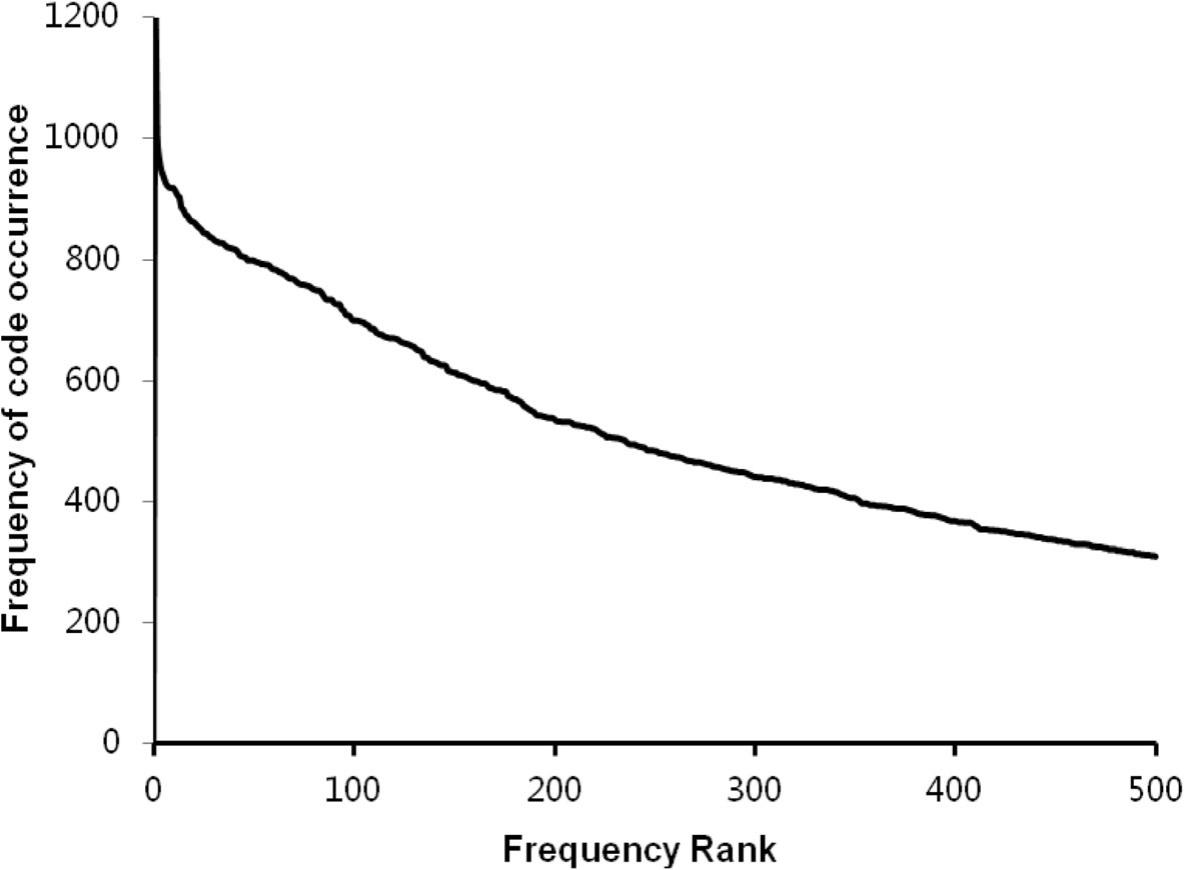
Distribution of the 500 high-frequency codes of the top 1000. Horizontal shows the other unmatched (in the first stage) 500 codes of the top 1000 from January 1, 2014 to April 30, 2018

In the second stage, the other unmatched (in the first stage) top 500 high-frequency codes from January 1, 2014 to April 30, 2018 are presented in Fig. 8 and in the Additional file 2. In fact, only 950 code categories could be generated automatically in our whole experiment, and the specific reasons will be described in the Discussion section. Table 4 shows the code categories that cannot be described by regexps. The monthly trends in coding quantity and a comparison between the amount of time needed for coders and the automatic coding system are presented in Fig. 6 and Fig. 7, respectively. Automatic coding in the second stage still has high precision and efficiency (Table 3).

**Table 4:** Codes of Uncorresponding the Description Models of the Regexps in the ICD-10

Codes	Diagnosis descriptions	Codes	Diagnosis descriptions
C13.901	Malignant neoplasm of hypopharynx, unspecified	R90.001	Intracranial space-occupying lession
C25.001	Malignant neoplasm of head of pancreas	S00.803	Superficial injury of face
C71.101	Malignant neoplasm of frontal lobe	S01.806	Open wound of face
C77.004	Secondary malignant neoplasm of supraclavicular lymph nodes	Z08.701	Follow-up examination after combined treatment for malignant neoplasm
C79.806	Secondary malignant neoplasm of neck	Z08.101	Follow-up examination after radiotherapy for malignant neoplasm
C79.826	Secondary malignant neoplasm of pelvis	Z08.202	Follow-up examination after chemotherapy for malignant neoplasm
C79.838	Secondary malignant neoplasm of blood vessels	Z08.001	Follow-up examination after surgery for malignant neoplasm
C83.302	Diffuse non-Hodgkin’s lymphoma of large cell	Z09.001	Follow-up examination after surgery for other conditions
D12.601	Benign neoplasm of colon, unspecified	Z47.002	Removal of internal fixation device for fracture
D12.801	Benign neoplasm of rectum	Z48.901	Surgical follow-up care, unspecified
D18.001	Intracranial hemangioma	Z51.005	Radiotherapy for malignant neoplasm of oesophagus
D18.037	Hemangioma of limbs	Z51.008	Radiotherapy for neoplasm of brain
D32.008	Benign neoplasm of cerebral meninges of frontal lobe	Z51.013	Radiotherapy for malignant neoplasm of cervix
D33.305	Benign neoplasm of auditory nerve	Z51.109	Chemotherapy for neoplasm of brain
D48.003	Nneoplasm of bone (uncertain or unknown behaviour)	Z51.118	Chemotherapy for malignant neoplasm of pancrea
D73.002	Asplenia, postsurgical	Z51.125	Chemotherapy for sarcoma
F09 01	Organic mental disorder of brain	Z51.801	Immunotherapy for neoplasm
G96.103	spinal meningeal cyst, unspecified	Z51.802	Symptomatic treatment for neoplasm
I69.801	Sequelae of cerebrovascular disease, unspecified	Z90.003	Acquied absebce of skull
K22.902	Neoplasm of oesophagus, unspecified	Z90.402	Acquied absebce of stomach
K63.901	Neoplasm of colon, unspecified	Z93.301	Colostomy status
K86.901	Pancrea space-occupying lesion, unspecified	Z95.002	Status following coronary stent implantation
M51.302	Intervertebral disc degeneration	Z96.601	Status following artificialjoint replacement
M84.491	Pathological fracture, not elsewhere classified	Z98.818	Postsurgical states of malignant neoplasm of brain
N63 01	Lump in breast, unspecified	Z98.820	Postsurgical states of malignant neoplasm of prostate

## Discussion

To our knowledge, this study was the first to develop and apply regexps in automatic coding, with the specific purpose of improving coding quality and efficiency. We constructed the description models of the regexps and inserted them into the coding system via the Oracle software. The automatic ICD-10 coding system completed more than 160,000 codes in 16 months, which reduced the workload of coders and showed high precision and efficiency.

Figure 3 indicates that the code categories are concentrated in the top 100 and that perfecting the corresponding description models of the regexps can reduce the number of FNs to improve the R values. Figure 8 shows that the difference of the frequency and variation range between the codes is not as large as Fig. 3, which is the main reason we only study the top 1000. Figures 4 and 5 show that diseases of the digestive system and circulatory system in our hospital are the most diverse and largest in number, which indicates that these two kinds of diseases need more attention in the process of building the description models of the regexps. In addition, class Z diseases are the second largest because our hospital has a large neoplasm treatment centre, involving many special screening examinations (Z12), follow-up examinations after treatment (Z08) and radiotherapy and chemotherapy sessions (Z51) for neoplasms. The curve A in Fig. 6 represents the diagnosis codes correctly assigned by the automatic coding system in every month of the two testing stages. Despite the downward trend in the first testing stage, in every month, the distances between the curve A and B remained stable; that is, the number of TPs were stable. The quantity of automatic coding decreased because of changes in hospital management, resulting in the system not running for some days. Figure 7 shows the time needed for automatic coding takes nearly 100 times less than manual coding, which clearly presents automatic coding can save much time.

The values of P for the first and second test stages were up to 89.27 and 88.38%, respectively. However, two main factors result in low R, F, and A values. First, automatic coding can only be executed when the programmer starts the program. Currently, it can only be run twice a day: starting working in the morning (8:00 am) and in the afternoon (14:30). Because clinicians usually complete the homepage of the discharge medical records at the end of their work, the number of diagnosis descriptions waiting to be coded peaks at these two times. Starting the program at these times can realize the value of automatic coding very well. At the same time, the coders are also manually coding. When the program stops, these diagnosis descriptions that should be automatically coded are actually completed by the coders. This leads to too many FNs. The more FNs, the smaller the R value is, and the smaller the R value is, the smaller the F value. Second, of more than 8000 code categories in our hospital, we only matched 950 code categories with high frequency, that is about 7000 code categories with frequencies below 300 have been lost. Table 3 shows the unmodeled code categories produced about 300,000 missing codes in 16 months from 10/1/2017 to 1/31/2019, which made the number of TNs large. The high negative values correspond to the low positive values; that is, the accurately assigned codes are few, and the A values are relatively low. Nevertheless, the values of R, F and A increased in the second testing stage, which illustrated that expanding the total number of matching codes was effective. Table 4 shows that the corresponding description models of the regexps failed to establish 50 code categories (on the top 1000), which were mainly concentrated in factors influencing health status and contact with health services (class Z) and neoplasms (classes C and D). In addition, other code categories are unspecified. The main reason for this result is that diagnosis descriptions recorded by clinicians are not standardized and vary greatly for these diseases, so the correct diagnosis cannot be coded until the coders consult the complete electronic medical record. The results suggest that clinicians need to strengthen their standardization of diagnosis descriptions when recording diagnoses, especially for classes C, D and Z diseases, while programmers and coders should spend more time on these diseases when building models. On the whole, our system has high precision. With the participation of programmers, clinicians and coders, the accuracy of the system can be improved by focusing on the high-frequency diseases and code categories and repeatedly improving the quality and quantity of regexps.

In recent years, although many studies have focused on automatic ICD coding, we want to highlight the following advantages presented by our study. First, compared to other theoretical studies on model validation using public databases [29–31], we use our hospital data for research to make a system that can be directly applied to practical work. Second, coders could identify their own shortcomings and strengthen communication with clinicians in the audit process to improve their accuracy. Third, our hospital receives a large number of doctors for standardized trainings and refreshers every year. Our doctors record diagnosis descriptions in a variety of ways, so our description models of the regexps have strong representativeness and applicability. Fourth, the regexps represent rules that can be easily understood by workers, which requires less involvement of experts in system implementation and can improve the applicability to small-scale medical institutions with more limited information technology. Five, we update the existing manual coding system based on the rule base of regexps to reduce workload and improve the work quality of coders. The technical requirements and computational cost are less than those of the other methods found in most studies [7, 11], [32–36]. CNN [18, 34–36] is one of the state of the art proposals to solve the problem of automatic ICD coding. Despite their high accuracy, there is still a long way to go before they can be used in practice. Our automatic coding system has been running steadily, which can solve the main problems faced by most medical institutions at present - large amount and repetitive coding. Our system is designed and completed in a relatively short time by our own programmers, which runs in a simple environment. Unlike the complex methods described above, they often require the assistance of engineers of information company. The description models of regexps, we have established have good representativeness and can be used for reference. Overall, our method can transfer to other institutions. Programmers can modify these regexps slightly according to actual situation and write them into existing coding system to run.

There are also shortcomings in our study. First, the automatic coding program runs twice a day: once in the morning and once in the afternoon. When the program is not running, coders are required to do manually input the codes. The next step of our study is to explore how to automatically code the diagnosis immediately after the clinician completes the records. Second, coders are required to perform the last step of auditing, so only semi-automation can be achieved. Code auditing puts forward higher requirements for the ability of coders, and coders should continue to participate in relevant professional training and learning. Standardized diagnosis descriptions are beneficial to improve the correctness of coding. The ICD-10 classification data of some error-prone codes can be sent to the relevant clinical departments, which arouses the attention of clinicians to the standardized writing of discharge diagnosis descriptions. Whether a gold standard can be established for automatic coding auditing remains to be studied. Third, it is hard to build the description models of the regexps for identical diseases with too different diagnosis terms. Our study is based on the diagnosis of common diseases (the top 1000) and fails to include uncommon diseases. Therefore, in future work, with the complete ICD-10 coding set as the goal, matching rules need to be improved constantly. In addition, the recall, F-measure and accuracy are low in our study compared to these method mentioned above [34–36]. For example, the CNN based method had reached a F-measure of 60.86% with high efficiency [34], and the reference [36] building a feature matrix, by a pretrained word embedding model used to train a CNN had a high testing accuracy (F-measure 90.86%). Whether our system can be fully automated with high precision by combining with the state of the art is a long-term task that we need to consider.

## Conclusion

The description models of the regexps can be used to match diagnosis descriptions and ICD codes, which are well-suited for the coding task. The proposed upgraded coding system is feasible and practical for the automatic coding of ICD-10 codes. Further studies are warranted to perfect the description models of the regexps and to develop synthetic approaches to improve system performance.

## Supplementary information

**Additional file 1.**

**Additional file 2.**

## Data Availability

All data generated or analyzed during this study are included in the Additional file 2.

## Abbreviations

ICD: International Statistical Classification of Diseases and Related Health Problems
CCD: Chinese Version of Classification and Codes of Diseases
DRG: Diagnosis-related group
DRG-PPS: Diagnosis-related group-based prospective payment system
SVM: Support vector machine
CCN: Convolutional neural network
NLP: Natural language processing
CCN: Convolutional neural network
regexps: regular expressions
TP: True positive
FP: False positive
FN: False negative
TN: True negative
