# Supplementary material for: Construction of a semi-automatic ICD-10 coding system
Source: BMC Med Inform Decis Mak. 2020 Apr 15;20:67. doi: 10.1186/s12911-020-1085-4 (PMC7157985; doi:10.1186/s12911-020-1085-4)
Supplement: Supplementary file 1 — Additional file 1. [file 12911_2020_1085_MOESM1_ESM.doc]

Procedural codes:

PARENT.st_3.Text = 'checking, please wait......'

PARENT.st_3.Visible = TRUE

li_cc = PARENT.dw_cc.Retrieve(gi_jg)

IF li_cc > 0 THEN

FOR j = 1 TO li_cc

ls_pid1 = PARENT.dw_cc.Object.patient_id[j]

li_vid1 = PARENT.dw_cc.Object.visit_id[j]

ls_dtype = PARENT.dw_cc.Object.diagnosis_type[j]

li_dno = PARENT.dw_cc.Object.diagnosis_no[j]

DELETE FROM diagnosis_category_log WHERE patient_id = :ls_pid1 AND visit_id = :li_vid1

AND diagnosis_type = :ls_dtype AND diagnosis_no = :li_dno

USING sqlca;

IF sqlca.SQLCode = 0 THEN

CONTINUE

ELSE

ROLLBACK USING sqlca;

END IF

NEXT

IF sqlca.SQLCode = 0 THEN

COMMIT USING sqlca;

PARENT.dw_cc.Retrieve(gi_jg)

END IF

END IF

PARENT.st_3.Text = 'Coding, please wait......'

PARENT.st_3.Visible = TRUE

ll_rows = PARENT.dw_3.Retrieve(gi_jg)

IF ll_rows > 0 THEN

PARENT.st_3.Text = ' Codes is being saved, please wait......'

SELECT sysdate INTO :ld_today FROM dual;

FOR i = 1 TO ll_rows

ls_pid = PARENT.dw_3.Object.patient_id[i]

li_vid = PARENT.dw_3.Object.visit_id[i]

ls_diag_type = PARENT.dw_3.Object.diagnosis_type[i]

li_diag_no = PARENT.dw_3.Object.diagnosis_no[i]

ls_diag_desc = PARENT.dw_3.Object.diagnosis_desc[i]

ls_diag_code = PARENT.dw_3.Object.diagnosis_code[i]

ll_insert_row = PARENT.dw_2.InsertRow(0)

PARENT.dw_2.Object.patient_id[ll_insert_row] = ls_pid

PARENT.dw_2.Object.visit_id[ll_insert_row] = li_vid

PARENT.dw_2.Object.diagnosis_type[ll_insert_row] = ls_diag_type

PARENT.dw_2.Object.diagnosis_no[ll_insert_row] = li_diag_no

PARENT.dw_2.Object.diagnosis_code[ll_insert_row] = ls_diag_code

ll_insert_row1 = PARENT.dw_log.InsertRow(0)

PARENT.dw_log.Object.ddate[ll_insert_row1] = ld_today

PARENT.dw_log.Object.patient_id[ll_insert_row1] = ls_pid

PARENT.dw_log.Object.visit_id[ll_insert_row1] = li_vid

PARENT.dw_log.Object.diagnosis_type[ll_insert_row1] = ls_diag_type

PARENT.dw_log.Object.diagnosis_no[ll_insert_row1] = li_diag_no

PARENT.dw_log.Object.diagnosis_desc[ll_insert_row1] = ls_diag_desc

PARENT.dw_log.Object.diagnosis_code[ll_insert_row1] = ls_diag_code

NEXT

PARENT.st_bcbm.Text = String(ll_rows) + 'number'

IF PARENT.dw_log.Update() = 1 THEN

IF PARENT.dw_2.Update() = 1 THEN

COMMIT USING sqlca;

PARENT.st_3.Visible = FALSE

IF gs_send = '1' THEN

uf_httpsend(gs_phone,String(ld_today,'yyyy-mm-dd') + ':coding success!total'+String(ll_rows)+'number')

END IF

sleep(10)

HALT CLOSE

ELSE

PARENT.st_3.Text = 'coding failure! checking and recoding! '

sleep(10)

HALT CLOSE

END IF

ELSE

PARENT.st_3.Text = ' coding failure! checking and recoding!'

sleep(10)

HALT CLOSE

END IF

ELSE

SELECT sysdate INTO :ld_today FROM dual;

IF gs_send = '1' THEN

uf_httpsend(gs_phone,String(ld_today,'yyyy-mm-dd') + ': no new codes!')

ELSEIF gs_send = '0' THEN

MessageBox(' system prompt ',' no new codes!')

END IF

PARENT.st_3.Text = ' no new codes!'

sleep(10)

HALT CLOSE

END IF

SQL statements:

Select a.patient_id,a.visit_id,a.diagnosis_type,a.diagnosis_no,a.diagnosis_desc,b.dcode diagnosis_code

from (

select a.patient_id,a.visit_id,a.diagnosis_type,a.diagnosis_no,a.diagnosis_desc

from diagnosis a,(

select patient_id,visit_id,diagnosis_type,diagnosis_no

from diagnosis

where (patient_id,visit_id) in (

select a.patient_id,a.visit_id

from diagnosis a

where (a.patient_id,a.visit_id) in (select patient_id,visit_id from pat_visit where to_char(discharge_date_time,'yyyymmdd') >= to_char(sysdate - :jg,'yyyymmdd')

and to_char(discharge_date_time,'yyyymmdd') <= to_char(sysdate,'yyyymmdd'))

and a.diagnosis_type = '3'

group by a.patient_id,a.visit_id

)

and diagnosis_type in ('1','2','3','A')

minus

select a.patient_id,a.visit_id,a.diagnosis_type,a.diagnosis_no

from diagnostic_category a

where (a.patient_id,a.visit_id) in (select patient_id,visit_id from pat_visit where to_char(discharge_date_time,'yyyymmdd') >= to_char(sysdate - :jg,'yyyymmdd')

and to_char(discharge_date_time,'yyyymmdd') <= to_char(sysdate,'yyyymmdd') )

group by a.patient_id,a.visit_id,a.diagnosis_type,a.diagnosis_no

) b

where a.patient_id=b.patient_id and a.visit_id=b.visit_id and a.diagnosis_type=b.diagnosis_type and a.diagnosis_no=b.diagnosis_no

) a,diagnosis_vs_category b

where regexp_like(a.diagnosis_desc,b.ddesc,'i')

order by a.patient_id,a.visit_id,a.diagnosis_type,a.diagnosis_no
